# Emerging roles of a pivotal lncRNA SBF2-AS1 in cancers

**DOI:** 10.1186/s12935-021-02123-3

**Published:** 2021-08-09

**Authors:** Qian Lu, Jun Lou, Ruyun Cai, Weidong Han, Hongming Pan

**Affiliations:** 1 Department of Medical Oncology, Sir Run Run Shaw Hospital, College of Medicine, Zhejiang University, Hangzhou, China; 2 Department of Colorectal Surgery, Sir Run Run Shaw Hospital, College of Medicine, Zhejiang University, Hangzhou, China

**Keywords:** LncRNA, SBF2-AS1, Cancers, Molecular mechanism, Biomarker, Therapy target

## Abstract

Long non-coding RNAs refer to transcripts over 200 nt in length that lack the ability to encode proteins, which occupy the majority of the genome and play a crucial role in the occurrence and development of human diseases, especially cancers. SBF2-AS1, a newly identified long non-coding RNA, has been verified to be highly expressed in diversiform cancers, and is involved in processes promoting tumorigenesis, tumor progression and tumor metastasis. Moreover, upregulation of SBF2-AS1 expression was significantly related to disadvantageous clinicopathologic characteristics and indicated poor prognosis. In this review, we comprehensively summarize the up-to-date knowledge of the detailed mechanisms and underlying functions of SBF2-AS1 in diverse cancer types, highlighting the potential of SBF2-AS1 as a diagnostic and prognostic biomarker and even a therapeutic target.

## Background

The human genome consists of 2.86 billion nucleotide sequences, most of which can be transcribed, yet less than 2% of them possess translational potential [1–3]. Non-coding RNAs (ncRNA) make up the bulk of these transcripts, which can be divided into short ncRNAs represented by microRNAs (miRNAs) and long non-coding RNAs (lncRNAs) according to whether their length exceeds 200 nt [4]. LncRNAs are roughly annotated as transcripts longer than 200 nt and lack a positive-sense strand open reading frame longer than 100 amino acids [5]. Transcribed mainly by RNA pol II from intergenic or intron regions as well as gene-dense regions, lncRNAs are an extremely heterogeneous family, which is analogous to miRNA with 5′ capping and 3′ polyadenylation and splicing [6, 7]. Due to the advancement of sequencing technology and the integration of algorithms in recent decades [8–10], sufficient evidence has proven that lncRNAs play an important biological role in different diseases [11–13]. In terms of mechanism, existing studies have confirmed that lncRNAs act as signals, decoys, guides and scaffolds by interacting directly or indirectly with chromatin, proteins and other RNAs, and then exerting transcriptional and/or post-transcriptional regulatory activities in the nucleus and cytoplasm [14, 15]. The function of lncRNAs varies hinging on their subcellular localization [16]. Intranuclear lncRNAs are involved in the regulation of histone modifications, DNA methylation and chromatin remodeling, as well as interactions with chromatin modification complexes, transcription factors and proteins in the nucleus. Those located in the cytoplasm regulate genes mainly at the post-transcriptional and translational levels, including interactions with proteins in the cytoplasm, regulation of mRNA metabolism, and interactions with miRNAs as endogenous competitive RNAs (ceRNAs).

Cancer, as a global health problem, is the result of systemic or germline mutations in response to environmental factors; most of these mutations are located in regions with no coding potential, and most of their transcriptional products are lncRNAs [17, 18]. LncRNAs may act as oncogenes or antioncogenes, affecting tumorigenesis, progression, metastasis, and drug resistance by virtue of interactions with genes involved in the cell cycle, apoptosis, epithelial-mesenchymal transition (EMT), immunological response, phenotypic transformation, or other pluripotencies [19–24].

SET binding factor 2—antisense strand 1 (SBF2-AS1) is transcribed from the antisense strand of the SBF2 gene, which encodes myotubularin‐related protein 13 (MTMR13) [25, 26]. We obtained genomic information on SBF2-AS1 from the GeneCards database (https://www.genecards.org/). SBF2-AS1 is a lncRNA that is 2708 nucleotides in length and located on chromosome 11p15.4 (chromosome 11: 9758268-9811335). This transcript is localized in the cytoplasm, cytoskeleton, nucleus and extracellular regions. SBF2-AS1 is aberrantly expressed in cancers and is involved in carcinogenesis and tumor progression. Herein, we comprehensively reviewed research progress among cancers on the underlying mechanism and clinical significance of SBF2-AS1. Moreover, these provide support for the potential of SBF2-AS1 as a biomarker to predict prognosis and, more importantly, as a therapeutic target.

## Expression characteristics and mechanism of upregulation of SBF2-AS1 expression in tumors

**Fig. 1:**
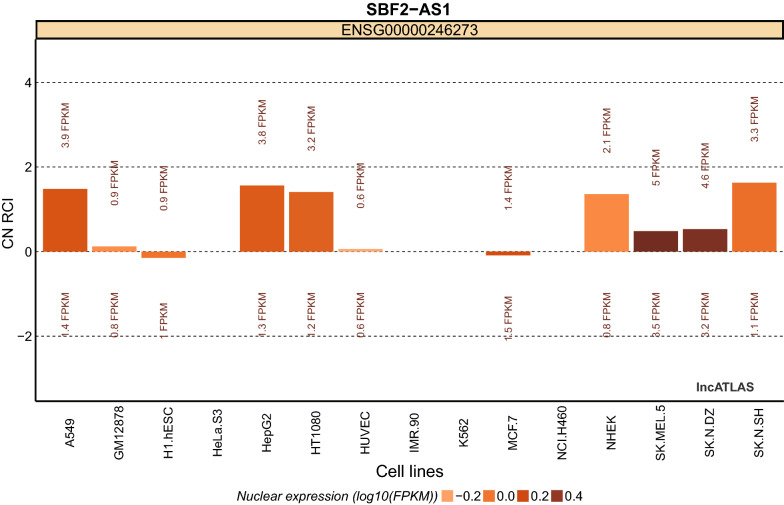
Cytoplasmic/nuclear Localization: RCI and expression values in cancer cells

LncRNA SBF2-AS1 was first characterized in non-small lung cancer (NSCLC) [26]. By analyzing a published lncRNA micromatrix dataset for NSCLC, Lv et al. found aberrant expression of SBF2-AS1. Subsequently, real-time quantitative PCR (RT-qPCR) analysis was performed on tumor tissues and adjacent normal tissues respectively, and the researchers found that SBF2-AS1 expression levels showed an approximately fivefold elevation on average in tumor tissues (in 36/41 patients). Then, more studies delved into the expression profile of SBF2-AS1 among other cancers, including pancreatic cancer (PC) [27], hepatocellular carcinoma (HCC) [28, 29], colorectal cancer (CRC) [30], gastric cancer (GC) [31], esophageal squamous cell cancer(ESCC) [32–34], breast cancer (BC) [35], glioblastoma (GBM) [36, 37], and others [38–41]. All of these studies with one accord showed that SBF2-AS1 was highly expressed in tumor tissue. Furthermore, RNA-FISH identified SBF2-AS1 was predominantly clustered in the cytoplasm [32, 42–44], which corresponds to its primary molecular mechanism. Additionally, we visualized the nuclear/cytoplasmic expression levels of SBF2-AS1 among diverse cancer cells through lncATLAS analysis (Fig. 1).

Nuclear transcription factors possess importance in the regulation of gene transcription via binding to a particular area of the promoter. It was shown that transcription factors are engaged in the upregulation of SBF2-AS1 expression. Yu et al. [36] found that the transcription factor nuclear factor of activated T-cells5(NFAT5), an aberrantly expressed transcription factor in tumors, can bind to the SBF2-AS1 promoter region at three presumptive sites through analyzing the JASPAR database, followed by PCR amplification and chromatin immunoprecipitation assays (ChIP) in glioblastoma cells. Finally, NFAT5 was shown to bind to and activate the promoter of SBF2-AS1 at the − 1453 to − 1448 bp region. Another research in glioblastoma revealed that transcription factor ZEB1 binds to the SBF2-AS1 promoter at the − 684 to − 676 bp region to augment SBF2-AS1 expression [37]. Analogously, Wang et al. demonstrated that E2F1 binds the SF2-AS1 promoter at the − 58 to − 66 bp region, thus upregulating SBF2-AS1 expression levels [38].

## Clinical significance of SBF2-AS1 in cancers

The expression of SBF2-AS1 is observably different between normal and tumor tissues, and it is significantly higher in tumor tissues. Kaplan–Meier survival analysis showed that patients in the high SBF2-AS1 expression group had worse overall survival (OS) than those with low SBF2-AS1 expression, except for those with clear cell renal cell carcinoma (ccRCC) (Table 1). In addition, univariate and multivariate regression analysis results indicated that SBF2-AS1 was an independent prognostic indicator of OS in multiple cancers [28, 45]. In LC tissues, clinicopathological characteristics that are associated with overexpression of SBF2-AS1 are larger tumor size, advanced tumor-node-metastasis (TNM) stage, more lymph node metastasis, distant metastasis, poor tissue type and poor histological differentiation [26, 42, 46, 47]. One study verified that the expression level of SBF2-AS1 is elevated in early-stage lung adenocarcinoma [48]. This finding suggested that early diagnosis of lung adenocarcinoma is possible, which is important in improving the prognosis of patients and prolonging patient survival. In HCC, SBF2-AS1 was notably correlated with lymph node metastasis, histologic grade, TNM stage, and vascular invasion. Likewise, the results of the analysis indicateed that a high level of SBF2-AS1 was linked to unfavorable clinical indices such as progressive TNM stages, distant metastasis and lymph node metastasis in certain cancers [27, 32, 35, 39, 44]. Briefly, SBF2-AS1 functions as an oncogene whose expression level is significantly correlated with undesirable clinicopathological features and poor OS, which bolsters up SBF2-AS1 as an excellent prognostic biomarker. Moreover, high expression of SBF2-AS1 supports its use as a diagnostic marker.

**Table 1:** Clinicopathologic significance of SBF2-AS1 in human cancers

Cancer type	Expression	Sample numbers	Clinicopathologic features	Refs.
Acute myeloid leukemia	Upregulated	173 cancer samples 70 normal samples	Overall survival	[38]
Breast cancer	Upregulated	50 paired samples	Lymph node metastasis, tumor size, clinical stage	[35]
Lung cancer	Upregulated	50 paired samples	Overall survival	[47]
		41 paired samples	Lymph node stage, TNM stage	[26]
		174 paired samples	Histological grade, lymph node metastasis, overall survival	[124]
		56 paired samples	TNM stage, lymph node metastasis, Overall survival	[50]
		35 paired samples	Age, tumor size, tumor tissue type, TNM stage, Overall survival	[46]
		30 normal samples 30 NSCLC samples 96 SCLC samples	Extensive stage, tumor size > 3 cm, lymph node metastasis, distant metastasis, overall survival	[125]
Osteosarcoma	Upregulated	45 paired samples	Tumor size, distant metastasis, ennking stage	[44]
Pancreatic cancer	Upregulated	82 paired samples	Degree of differentiation, TNM stage, lymph Node metastasis	[27]
Cervical cancer	Upregulated	66 paired	FIGO^a^ stage, lymph node metastasis, overall survival	[39]
Colorectal cancer	Upregulated	61 cancer samples 29 normal samples	TNM stages, survival rate	[30]
Papillary thyroid cancer	Upregulated	73 tumor samples (37 high and 36 low)	Overall survival	[40]
Glioblastoma	Upregulated	47 tumor samples 5 normal samples	Pathological grade	[36]
		20 primary samples 20 recurrent samples	Overall survival	[37]
Clear cell renal cell carcinoma	Upregulated	46 paired	–	[41]
Hepatocellular carcinoma	Upregulated	184 paired samples	Lymph node metastasis, histologic grade, TNM stage, overall survival	[28]
		134 paired samples	Vein invasion, TNM stage, overall survival rate	[29]
Gastric cancer	Upregulated	60 paired samples	TNM stage, overall survival	[31]
Esophageal squamous cell carcinoma	Upregulated	51 paired samples	Smoking and drinking history, tumor size, TNM stages, survival rate	[32]
		50 tumor samples 15 normal samples	Survival rate	[33]
		50 paired samples	Tumor size, survival rate	[34]

^a^International federation of gynecology and obstetrics

## Roles of SBF2-AS1 in diverse cancers

**Fig. 2:**
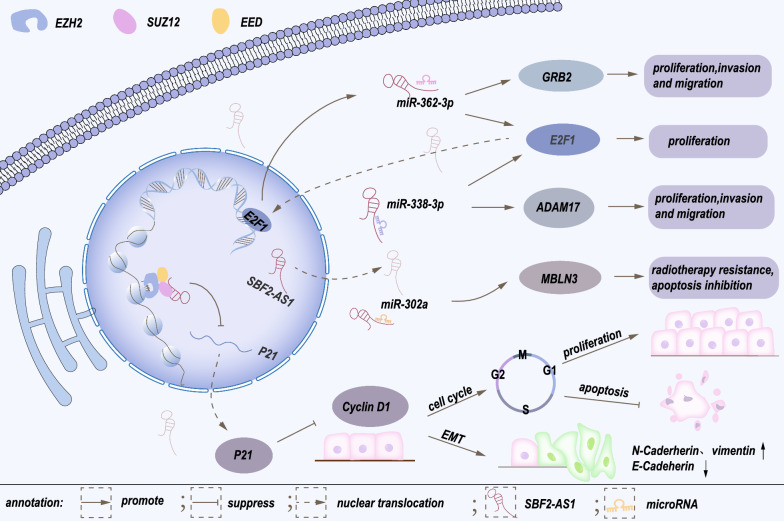
The regulatory mechanism of SBF2-AS1 in lung cancer. Upregulation of SBF2-AS1 expression could regulate lung cancer cell proliferation and invasion via different methods. SBF2-AS1 binds to subunits (EZH2 and SUZ12) of PRC2 to suppress the expression of P21 and augment cyclin D1 expression in NSCLC, thus promoting the cell cycle and EMT process. SBF2-AS1 facilitates NSCLC cell progression by regulating the miR-338-3p/ADAM17 axis. Moreover, SBF2-AS1 upregulated by E2F1 sponges miR-362-3p to promote GRB2 expression. Additionally, SBF2-AS1 enhances cell proliferation, migration and invasion via facilitating E2F1 through suppression of miR-362-3p/miR-338-3p

**Fig. 3:**
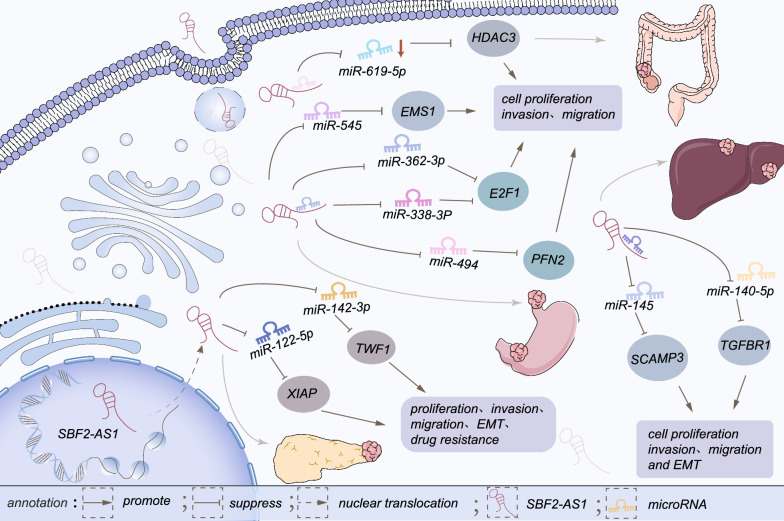
The regulatory mechanism of SBF2-AS1 in digestive system neoplasms. SBF2-AS1 promotes tumorigenesis and tumor progression in digestive tumors. In HCC, SBF2-AS1 promotes tumor development by regulating the miR-140-5p/TGFBR1 axis or miR-145/SCAMP3 network. In addition, cell proliferation, migration and invasion are enhanced via upregulating HDAC3 expression by inhibition of miR-619-5p in CRC. SBF2-AS1 promotes PC progression and amplifies drug tolerance by regulating the miR-122-5P/XIAP or miR-142-3p/TWF1 axis. SBF2-AS1 competitively binds to miR-338-3/miR-362-3p thus increasing E2F1 expression in ESCC cells.Additionally, SBF2-AS1 facilitated ESCC progress via regulating miR-494/PFN2 axis. By binding to miR-545, SBF2-AS1 promotes GC development and augments EMS1 expression

Extensive evidence suggests that dysregulation of SBF2-AS1 is pivotal in cancer cell proliferation, migration, invasion, apoptosis and EMT. Hereof, we elaborate the functions and underlying mechanisms of SBF2-AS1 in cancers (Table 2) and describe the mechanism of lung cancer and digestive system tumors (Figs. 2 and 3).

**Table 2:** Expression pattern and biological functions of SBF2-AS1 in human cancers

Cancer type	Expression	Biological Function	Related genes	Roles	Refs
Acute myeloid leukemia	Upregulated	Proliferation, Cell cycle, Apoptosis	miR-188-5p/ZFP91	Oncogene	[38]
Breast cancer	Upregulated	Proliferation, cell cycle, invasion, migration, apoptosis	miR-143/RRS1	Oncogene	[35]
Lung cancer	Upregulated	Promote cell proliferation, invasion, migration	SUZ12, p21, Cyclin D1	Oncogene	[26]
	Upregulated	Enhance radiotherapy resistance (promote cell proliferation, inhibit apoptosis)	miR-302a/MBNL3	Oncogene	[42]
	Upregulated	Promote cell proliferation, invasion and migration	miR-338-3p/ADAM17	Oncogene	[50]
	Upregulated	Promote cell proliferation, invasion, migration	E2F1/SBF2-AS1/miR-362-3p/GRB2	Oncogene	[46]
	Upregulated	Promote cell cycle and cell proliferation	miR-338-3p、miR-362-3p/E2F1	Oncogene	[48]
	Upregulated	Promote cell proliferation, inhibit apoptosis	FOXM1	Oncogene	[47]
Osteosarcoma	Upregulated	Proliferation, invasion, migration	miR-30a/FOXA1	Oncogene	[44]
Pancreatic cancer	Upregulated	Promote cell proliferation, invasion, migration and EMT, inhibit apoptosis	miR-142-3p/TWF1	Oncogene	[27]
	Upregulated	Promote cell proliferation, invasion and migration, inhibit apoptosis	miR-122-5p/XIAP	Oncogene	[43]
Cervical cancer	Upregulated	Proliferation, cell cycle, apoptosis	miR-361-5p/FOXM1	Oncogene	[39]
Colorectal cancer	Upregulated	Proliferation, invasion, migration	miR-619-5p/HDAC3	Oncogene	[30]
Papillary thyroid cancer	Upregulated	Proliferation, cell cycle,invasion, apoptosis	miR-431-5p/CDK14	Oncogene	[40]
Glioblastoma	Upregulated	Reduce temozolomide sensitivity, promote tumor cell DNA repair	Z1B1/SBF2-AS1/miR-151-3p/XRCC4	Oncogene	[37]
	Upregulated	Promote angiogenesis (enhance endothelial cells viability, migration and tube formation)	NFAT5/miR-338-3p/EGFL7/ERK	Oncogene	[36]
Clear cell renal cell carcinoma	Upregulated	Proliferation, invasion, migration, apoptosis	miR-338-3p/ETS1	Oncogene	[41]
Hepatocellular carcinoma	Upregulated	Promote cell proliferation, invasion, migration and EMT	miR-140-5p/TGFBR1	Oncogene	[28]
	Upregulated	Promote cell migration and invasion	miR-145-5p/SCAMP3	Oncogene	[57]
	Upregulated	Promote cell proliferation, invasion, migration and EMT	N-cadherin, vimentin	Oncogene	[29]
Gastric cancer	Upregulated	Proliferation, invasion, migration	miR-545/EMS1	Oncogene	[31]
Esophageal squamous cell carcinoma	Upregulated	Promote cell proliferation, invasion, migration and EMT, inhibit apoptosis	miR-494/PFN2	Oncogene	[33]
	Upregulated	Promote cell proliferation and inhibit apoptosis	miR-338-3p/miR-362-3p/E2F1	Oncogene	[34]
	Upregulated	Promote cell proliferation, invasion and migration	CDKN1A	Oncogene	[32]

### Lung cancer

As the second most prevalent and deadliest cancer worldwide, lung cancer (LC) is a health issue that needs to be addressed urgently. Non-small cell lung cancer (NSCLC) accounts for the majority of cases [49]. This disease is characterized by uncontrollable tumor proliferation, tumor heterogeneity, distant metastasis, tumor recurrence, and resistance to therapy. One choreographed in vitro experiment showed that SBF2-AS1 can bind to the subunit SUZ12, a core component of PRC2, through which it regulates enrichment of SUZ12 and trimethylation of histone 3 lysine 27 in the P21 promoter region. Loss-of-function experiment demonstrated that silencing of SBF2-AS1 remarkably upregulated P21 expression, thus suppressing NSCLC cell proliferation, migration and invasion [26].Additionally, one study manifested that SBF2-AS1 and a disintegrin and metalloproteinase 17 (ADAM17) share a functional miR response element, miR-338-3p, and SBF2-AS1 ultimately accelerates NSCLC progression through the ceRNA network [50]. Upregulation of SBF2-AS1 expression regulated E2F1 expression through sponging and repressing miR-338-3p or miR-362-3p in lung adenocarcinoma cells and contributed to cell proliferation [48]. Subsequently, Wang et al. proved that SBF2-AS1 directly binds miR-362-3p, further upregulating the expression level of growth factor receptor-bound protein 2 (GRB2) to promote NSCLC cell proliferation, migration and invasion [46]. Radiation tolerance is responsible for the poor response to radiotherapy. Yu et al. suggested that SBF2-AS1 plays an integral role in the radiosensitivity of NSCLC. Targeting the miR-302a/muscleblind-like splicing regulator 3 (MBNL3) signal axis, upregulated SBF2-AS1 expression distinctly strengthens NSCLC cell resistance to radiotherapy via reducing the expression of miR-302a [42]. Moreover, Qi et al. identified that SBF2-AS1 is linked to forkhead box protein M1 (FOXM1) via bioinformatics technology and Pearson correlation coefficients. Functionally, lncRNA SBF2-AS1 upregulates FOXM1 expression to inhibit apoptosis and promote proliferation of lung cancer cells [47]. Figure 1 summarizes the LncRNA SBF2-AS1 regulatory network in lung cancer.

### Hepatocellular carcinoma

Accounting for approximately 90% of total primary liver cancers, hepatocellular carcinoma (HCC) is a challenging disease worldwide with a dismal OS, which may be explained by its strong invasiveness and high recurrence rate [51, 52]. EMT, involved in different diseases, including cancers, refers to epithelial to mesenchymal cell transformation, through which cells are able to metastasize and invade [53]. Furthermore, diversified lncRNAs have been verified to modulate the EMT pathway in hepatocellular carcinoma [29, 54, 55]. Functional loss experiments observed that si-SBF2-AS1 markedly reduced the expression of N-cadherin and vimentin, but slightly restored the expression of E-cadherin, thereby attenuating the invasiveness and metastasis of HCC cells [56]. Consistent with previous studies, SBF2-AS1 served as a ceRNA to regulate the miR-140-5p/transforming growth factor beta receptor 1(TGFBR1) axis and thus promoted HCC cell EMT, cell proliferation, migration and invasion [28]. Besides, Zhou et al. indicated that SBF2-AS1 and secretory carrier membrane protein 3 (SCAMP3) levels were obviously upregulated in HCC. Combined with miR-145-5p, which is distinctly decreased, SBF2-AS1 and SCAMP3 form a regulatory network involved in the progression of HCC via bioinformatics analysis. However, further experimental attestation is required to clarify the mechanism of ceRNA [57].

### Esophageal squamous cell carcinoma

Esophageal squamous cell carcinoma (ESCC) accounts for 90% of esophageal cancers and has a high incidence in Asia. Despite advancements in diagnostic and therapeutic methods in recent years, the 5-year survival rate remains at approximately 15% [58, 59]. Several studies have indicated that SBF2-AS1 expression is increased, consequently facilitating ESCC cell proliferation, migration and invasion [32]. Functional experiments have shown that silencing SBF2‑AS1 upregulates cyclin-dependent kinase inhibitor 1A (CDKN1A) expression in ESCC, affceting cell cycle arrest and thereby restraining the proliferative ability of ESCC cells via arresting them at the G2 phase [32]. Furthermore, SBF2-AS1 directly inhibits miR-494, resulting in the increased expression of profilin 2 (PFN2) and accelerated tumor progression [33]. Zha et al. verified that SBF2-AS1 and miR-338-3P or miR-362-3p inhibit each other in ESCC cells, and emphasized that overexpression of SBF2-AS1 and E2F1 is involved in tumor development and apoptosis [45].

### Pancreatic cancer

Pancreatic cancer (PC) is a fatal malignancy worldwide [49]. For most PC cases, the 5-year survival rate does not exceed 10%, due to its asymptomatic characteristics [60, 61]. Drug resistance in pancreatic cancer also contributes to low survival rates in PC. Studies have shown that SBF2-AS1 expression is remarkably increased in PC cell lines [27, 43]. Besides, Hua et al. detected a noticeable increase in the expression level of SBF2-AS1 and twinfilin-1 (TWF1) in gemcitabine-resistant PC cells, while miR-142-3p expression was inversely downregulated. Deficiency in SBF2-AS1 expression regained the sensitivity of gemcitabine-resistant PC cells via repressing cell proliferation, migration, invasion, and EMT [27]. Both M2 macrophages and exosomes have been shown to be involved in certain phases during progression of tumors or drug resistance [62–64]. One study verified that exosomal lncRNA SBF2-AS1, which is derived from M2 macrophages of pancreatic cancer, regulates the level of X-linked inhibitor of apoptosis protein (XIAP) expression by integrating with miR-122-5p. Restricting the expression of SBF2-AS1 significantly elevated miR-122-5p level and inhibited XIAP expression, further inhibiting cell multiplication, and quenching invasiveness and metastasis in PC cells [43].

### Colorectal cancer

Colorectal cancer (CRC) possesses high morbidity and mortality rates on a global scale [49]. SBF2-AS1, as an oncogene, was identified to be overexpressed in CRC cell lines by Chen et al. In vitro experimental results showed that SBF2-AS1 positively regulates the expression of histone deacetylase 3 (HDAC3) in CRC cells via acting as a “sponge” of miR-619-5p and inhibiting its expression [30]. HDAC3, an essential subunit of the nucleosome remodeling deacetylase of the chromatin remodeling complex, has been certified to act as an oncogene in a wide range of tumors [65–67]. Functionally, upregulation of SBF2-AS1 expression can enhance HDAC3, therefore promoting proliferation, metastasis and invasiveness of CRC cells [30].

### Gastric cancer

Gastric cancer (GC), as a gastrointestinal tumor, is a malignancy that involves oncogenic activation, inactivation of tumor suppressor genes and aberrant protein expression [68–70]. He et al. indicated that SBF2-AS1 expression is significant increased in GC cell lines and plays a crucial role in tumor development. Forced upregulated SBF2-AS1 expression results in competitive binding of miR-545 through the ceRNA mechanism, thus increasing the expression of excess microsporocytes 1 (EMS1) and repressing miR-545 expression to facilitate GC cell growth and metastasis [31].

### Glioblastoma

Glioblastoma (GBM) is the most common primary malignant tumor of the brain, with invasive clinical characteristics and a frightening lethality rate [71, 72]. Even under a comprehensive treatment strategy that includes surgical resection, chemotherapy based on temozolomide (TMZ) and adjuvant radiotherapy, the recurrence of glioma is inevitable, resulting in a 3-year survival rate < 10% [73]. Glioblastoma, a highly vascularized tumor, has high degree of growth malignancy and depends on the formation of new blood vessels [74]. Previous studies have demonstrated that lncRNAs are involved in angiogenesis in glioblastoma [75, 76]. Yu et al. revealed that nuclear factor of activated T cells 5 (NFAT5) enhanced SBF2-AS1 expression at the transcriptional phase.Thereafter, SBF2-AS1 functions as a ceRNA, sponges miR-338-3p and alleviates the repressive effect on EGF like domain multiple 7 (EGFL7) at the post-transcriptional level, which stimulates endothelial cell activity, migration and tube formation. Further experiments demonstrated that EGFL7 exerts a potent angiogenic role through activating the ERK pathway [36]. In addition, transcription factor zinc finger E-box binding homeobox 1 (ZEB1) upregulates the level of X-ray repair cross complementing 4 (XRCC4) expression via the SBF2-AS1/miR-151-3p axis to enhance resistance to temozolomide in glioma cell lines. Highly expressed exosomal SBF2-AS1 was also shown to promote TMZ resistance in GBM cells in vivo [37].

### Cervical cancer

Cervical cancer (CC) is a prevalent gynecologic malignant tumor [49]. Even with advances in treatment, the five-year survival rate for CC remains frustratingly low, principally attributed to tumor metastasis and recurrence [77, 78]. Gao et al. indicated that SBF2-AS1 expression was prominently increased in CC cells and positively correlated with FOXM1 expression because SBF2-AS1, as ceRNA binding to miR-361-5p, mitigated the suppressive effect of miR-361-5p on FOXM1. Gain-of-function experiments verified that upregulation of SBF2-AS1 expression enhances cell proliferation, and inhibits apoptosis of CC cells [39].

### Acute myeloid leukemia

Acute myeloid leukemia (AML) is a malignant disease characterized by unregulated clonal propagation, abnormal infiltration and poor differentiation of immature myeloblasts of the hematologic system [79]. AML is a heterogeneous malignancy that is accompanied by cytogenetic changes, various biological features and molecular abnormalities [80, 81]. Dysregulation of lncRNAs is one of the genetic alterations [82, 83]. SBF2-AS1 was identified to be elevated in AML cells, and contributed to AML development via triggering cell cycle arrest and apoptosis. Further experiments indicated that SBF2-AS1 functions as a ceRNA to alter the expression level of zinc finger protein 91(ZFP91) by suppressing the expression of miR-188-5p [38].

### Breast cancer

Breast cancer (BC) is the most common malignant tumor among global population and has a high mortality rate second to that of lung cancer [49]. Although there are various treatment options for breast cancer, choosing an appropriate therapy method for highly invasive and metastatic cases is still a major challenge. Previous studies have suggested that lncRNAs may offer new treatment targets for breast cancer patients [84–86]. Xia et al. proved that SBF2-AS1 expression and ralstonia solanacearum 1 (RRS1) expression are positively correlated. SBF2-AS1 has been proven to sequester miR-143 and subsequently increase the expression of RRS1 in BC cells, thereby repressing BC cell apoptosis and promoting cell proliferation, migration and invasion [35].

### Osteosarcoma

Osteosarcoma is a malignant bone tumor and one of the most common sarcomas [87]. This malignancy originates from primitive osteogenic mesenchymal cells and occurs mostly in children, adolescents and young people. Multiple alternative strategies developed in the past several decades have not succeeded in improving the prognosis of patients with osteosarcoma [88]. Recently, scholars have focused on SBF2-AS1 as a therapeutic target in osteosarcoma. A study indicated that the level of SBF2-AS1 is remarkably elevated, and this molecule positively regulates forkhead box A1 (FOXA1) expression via sponging miR-30a and suppressing the expression of miR-30a in osteosarcoma cells, significantly affecting biological processes, such as cell multiplication, invasion, migration and apoptosis [44].

### Papillary thyroid cancer

As a prevalent endocrine-system malignant neoplasm, papillary thyroid cancer (PTC) accounts for the majority of thyroid cancer [89]. The biological characteristics of PTC are highly diverse, ranging from nonprogressive to invasive metastatic carcinoma. Although there are various treatment modalities available for papillary thyroid cancer, researchers are still searching for an optimal treatment [90, 91]. Wen et al. found that SBF2-AS1 expression increased distinctly in PTC cells and strongly affected tumor progression. Functionally, downregulation of SBF2-AS1 expression decreased cyclin-dependent kinase 14(CDK14) expression and conversely increased miR-431-5p expression, which suppresses PTC cell viability and promotes apoptosis [40].

### Clear cell renal cell carcinoma

Clear cell renal cell carcinoma (ccRCC) as the dominant type of kidney cancer, has the highest fatality rate compared to other types [92]. It was reported that SBF2-AS1, serving as a molecular sponge of miR-338-3p, competitively binds to miR-338-3p and inhibits its expression, thus enhancing ETS1 expression in ccRCC cells. Further functional studies showed that decreased expression of SBF2-AS1 restrains the proliferation and invasiveness of ccRCC cells and triggers apoptosis and autophagy by blocking ETS1 and upregulating miR-338-3p expression [41].

## Regulatory mechanism of downstream genes by SBF2-AS1

### Interaction with RNA (as a ceRNA)

The ceRNA hypothesis reveals a mechanism of RNA interactions that has been useful in explaining abnormal transcriptome changes. The hypothesis presumes that ceRNAs, including circRNAs, lncRNAs, and mRNAs, regulate downstream gene expression by competitively binding microRNAs. The novel lncRNA SBF2-AS1, with a length of 2708 bp, primarily localized in the cytoplasm, possesses many latent miRNA binding sites and exerts an effect on malignant biological processes principally through a ceRNA mechanism. It was also observed that the expression level of SBF2-AS1 was mostly higher than or equivalent to that of miR-338-3p, which sustains its roles in cancers.

For instance, Yang et al. [41] identified that SBF2-AS1 was located in the cytoplasm by FISH, followed by a dual luciferase reporter gene assay, which showed that the luciferase activity of the vector carrying SBF2-AS1-WT was markedly weakened in 768-O cells by miR-338-3p mimics. Later, an RNA binding protein immunoprecipitation (RIP) assay was applied to verify that both miR-338-3p and SBF2-AS1 were enriched in beads containing Ago2. To further illuminate the interaction between SBF2-AS1 and miR-338-3p, through RNA pull-down, the researchers detected a noticeable rise in the concentration of SBF2-AS1 in the bio-miR-338-WT group. By RT-qPCR assays, elevated miR-338-3p expression was detected after treatment with either miR-338-3p mimics or si-SBF2-AS1. In contrast, the miR-338-3p expression level was significantly decreased after using PCDNA-SBF2-AS1 or miR-338-3p inhibitor. From this, it can be concluded that SBF2-AS1 functions as a ceRNA by sponging miR-338-3p and negatively regulates its expression in clear cell renal cell cancer. SBF2-AS1 was also observed to interact with miR-338-3p similarly in NSCLC and glioblastoma.

Based on the results of the dual luciferase reporter gene assay, Ago2-RIP assay, RNA pull-down and RT-qPCR, SBF2-AS1 can serve as a sponge that competitively binds to miR-122-5p, miR-362-3p, miR-188-5p, miR-30a, miR-142-3p, miR-361-5p, miR-151-3p and so on (Table 2), thus regulating biological processes in tumor cells. Bioinformatics analysis is increasingly valued by researchers as an aid to tumor studies. In particular, these methods have shown potential in the analysis of RNA-RNA interactions. By performing bioinformatic analysis, Zhou et al. identified miR-145 binding site of SBF2-AS1; however, further experiments are needed to verify whether the putative binding site is functional.

### Interaction with DNA

SBF2-AS1 engages in methylation modification of genes to regulate their expression at the DNA level [26, 32]. Polycomb repressive complex 2 (PRC2) is a chromatin-associated methyltransferase involved in catalyzing the methylation of lysine 27 on histone H3 (H3K27) [93], which is an important regulator of tumor development and progression. Previous studies have demonstrated that lncRNAs may function in regulating PRC2 recruitment [94] or directly bind to its subunits, such as SUZ12 [95], thus regulating epigenetic modification. SBF2-AS1 can recruit and then bind SUZ12 and EZH2 of PRC2, then enrich PRC2 in the promoter region of P21, thus inactivating P21, which arrests the cell cycle at S phase to promote indefinite proliferation in NSCLC as well as ESCC.

### Molecular interference between SBF2-AS1 and the signal pathway

SBF2-AS1 mainly acts as a ceRNAs to regulate downstream genes of miRNAs, many of which are involved in signaling pathways. For example, LncRNA SBF2-AS1 can regulate the ERK signaling pathway [36]. Researches have demonstrated that ERKs and their signaling pathways play a role in mediating and amplifying signals during tumor invasion and metastasis by regulating cell proliferation, differentiation and viability [96]. Overexpression of SBF2-AS1 can upregulate EGFL7 expression and thus significantly increase the expression of p-ERK, which is an important member of the MAPK/ERK signaling pathway and one of the key components to initiate the signal cascade [97]. The ERK signaling pathway proteins include RAS, RAF, MEK, MAPK, STAT3, p90rsk, cFos, c-Myc and n-Myc. However, the specific molecules through which SBF2-AS1 exerts its oncogenic effects need to be further investigated.

In addition, SBF2-AS1 show crosstalk with other signaling pathway members; for example, GRB2, a key adapter protein that activates the Ras/Erk pathway, is involved in EGFR-mediated signaling transduction [98]. Upregulated Grb2 expression correlates with p-c-Raf, p-Mek1/2 and p-Erk1/2, which are pivotal members of the Ras/Erk pathway [99]. Another example is HDAC3, which is increased in CRC by overexpression of SBF2-AS1and is reported to be involved in TGF-β signaling pathway [100]. HDAC3 is positively correlated with the regulation of TGF-β1, p-smad2, and p-smad3, all of which are TGF-β-related proteins. Multiple regulatory pathways exist in the same tumor, even containing the same molecules of action, but the interactions and relationships between SBF2-AS1 and regulatory pathways still require further molecular experimental studies.

## SBF2-AS1 as a therapeutic target for cancers

Since lncRNA expression exhibits striking tissue specificity compared with mRNA expression [101], an increasing number of studies have shown that targeting lncRNAs is a desirable therapeutic approach [102]. As previously noted, lncRNA SBF2-AS1, as a novel oncogene, plays a critical role in tumor progression and is associated with poor OS. More importantly, the core role of SBF2-AS1 is a shared feature of plentiful tumors. All the above findings suggest that silencing SBF2-AS1 is an effective therapeutic strategy to inhibit tumor growth. Besides, SBF2-AS1 binds to miRNAs as a ceRNA and abolishes the inhibitory effect on downstream target genes. Thus, upregulation of miRNA expression to quell the action of downstream target genes can be regarded as a therapeutic strategy. In recent decades, RNA-based anticancer therapies have gradually moved from concept to reality [103–105]. Compared to targeting proteins, lncRNA-targeting drugs exhibit preferabletherapeutic potential because they bypass the pleiotropic effects of many current therapeutic modalities [106]. Current prevalent strategies for lncRNA targeting include antisense oligonucleotides (ASOs), clustered regularly interspaced short palindromic repeats/CAS9 (CRISPR/CAS9), and small molecules [107, 108]. ASOs, chemically synthesized short fragment oligonucleotides, bind to RNA through Watson–crick base pairing, thereby altering gene expression [109]. Currently, ASOs targeting different mRNAs have shown clinical benefits for specific diseases [110]. These molecules show effective targeting of lncRNAs in the preclinical stage [111–113]. One study noted that ASOs targeting and silencing MALAT1 contributed to moderate tumor proliferation in the MMTV (mouse mammary tumor virus)-PYMT mouse breast cancer model, along with remarkable differentiation into cystic tumors and a decrease in lung metastasis [113]. Based on these findings, we believe that the ASO product targeting SBF2-AS1 is promising for tumor therapy in the future. CRISPR/Cas9, a gene editing technology, targets the region of the gene promoter to silence transcription and has been used in cancer cells and animal cancer models [114–116]. A choreographed animal study of gastric cancer revealed that targeting lncRNA GMAN by CRISPR-CAS9 inhibited tumor metastasis and improved survival rates in mice [115]. By this means, we were able to achieve silencing the transcription of SBF2-AS1 expression loci. Notably, small molecules have recently become a focus of research by virtue of their nonimmunogenic nature, systemic delivery and highly plastic physicochemical structure [117]. An RNA-targeted small molecule, risdilasm, was approved for the treatment of spinal muscular atrophy in 2020 in the USA [118]. As small molecules targeting lncRNAs have not yet been druggable, studies have increasingly focused on breaking through this dilemma [119–121]. YK-4-279, a novel man-made molecule, displays a strong suppressive impact on Ewing sarcoma cells via regulating lncRNA HULC (highly upregulated in liver cancer) [121]. By drawing on these experiences, we may be able to design small molecules that precisely target SBF2-AS1 based on its secondary structure and mode of action for clinical treatment. In conclusion, these modalities are not yet mature and lncRNA-based therapeutics are in the preliminary period of development, requiring more intensive and in-depth research as well as clinical trials to support them and accelerate their development.

## Discussion

In this review, we present a comprehensive analysis of all the studies on SBF2-AS1 to date, describing the clinical studies as well as molecular mechanisms and biological functions of SBF2-AS1 in terms of tumor classification, followed by an in-depth discussion of the molecules that interact with SBF2-AS1. The biological functions of SBF2-AS1 are mainly related to tumor proliferation, invasion, metastasis, EMT, angiogenesis, chemoresistance and radiosensitivity. The current clinical studies on SBF2-AS1 are mainly concerned with intertissue variation in expression and correlation with some clinical features, which are not sufficient to support it as a diagnostic biomarker and indicate the need for more accurate, detailed and in-depth large clinical trials to confirm its role.

Current studies all suggest a consistent role for SBF2-AS1 in tumors. LncRNA SBF2-AS1, which acts as an oncogene, is overexpressed in various tumor tissues and cells, including lung cancer, hepatocellular carcinoma, esophageal squamous cell carcinoma, breast cancer, ovarian cancer, cervical cancer, osteosarcoma, colorectal cancer, and pancreatic cancer. Moreover, studies have revealed that SBF2-AS1 expression is detected to be upregulated in exosomes, which is correlated with the sensitivity of GBM cells to temozolomide and with tumor progression in PC. In addition, SBF2-AS1 exerts an impact on drug resistance in CRC and radioresistance in NSCLC. Elevated levels of SBF2-AS1 reinforce the tolerance to radiotherapy in NSCLC. Upregulation of SBF2-AS1 expression in CRC is related to resistance to gemcitabine. In-depth clinical analysis showed that there exists an intimate connection between the expression level of SBF2-AS1 and the clinical features of patients. Upregulation of SBF2-AS1 expression predicted poor OS, and was associated with larger tumor size, advanced TNM stage, lymph node metastasis, and poorer pathological differentiation. Mechanistically, SBF2-AS1 predominantly forms a ceRNA network with miRNAs and downstream genes to regulate tumor progression. There are different regulatory axes in the same tumor, such as enhancing ESCC cell proliferation through miR-188-5p/PFN2 or interacting with miR-338-3P/miR-362-3p to activate E2F1 expression. These phenomena may be mediated by the presence of several different miRNA binding sites in the SBF2-AS1 sequence. In addition, different tumors may activate different downstream genes through the same miRNA molecule, for example, in ccRCC, by sponging miR-338-3p and upregulating ETS1 expression or in NSCLC via activating ADAM17, thus promoting tumor proliferation, invasion and metastasis, which is due to the functional characteristics of miRNAs capable of regulating multiple downstream genes. These studies alone have not fully elucidated and strongly support the roles of SBF2-AS1 in cancers, and more experimental research is needed. More importantly, whether lncRNA SBF2-AS1 functions as a sponge of microRNAs should be carefully assayed in each case, and particularly the relative expression of the lncRNA and microRNAs should be quantified to make any conclusion. Furthermore, SBF2-AS1 may regulate the MAKP/ERK, the TGF-β signaling pathways. The interactions between SBF2-AS1 and other signaling pathways require more meticulous experiments for confirmation. Regarding the upstream regulatory mechanism of SBF2-AS1, existing studies have confirmed that high expression of SBF2-AS1 is controlled by the transcription factors E2F1, ZEB1 and NFAT5 at the transcriptional level. Whether SBF2-AS1 is subject to epigenetic regulation and splicing is not yet known, and further researches are requisite to figure out.

### Perspectives

The regulatory mechanisms of lncRNAs are diverse and complex. SBF2-AS1 has already been reported to promote cancer development, chemoresistance and radio-resistance by interacting with RNA, DNA or crosstalk with molecules of various signal pathways. Molecular and clinical researches of SBF2-AS1 are still in their infancy, and SBF2-AS1 shows potential for future applications. First, directly or indirectly targeting SBF2-AS1 to suppress its expression through ASOs, CRISPR/CAS9 or biological molecules may be a viable remedy for cancers as mentioned above, in the future, especially for some current refractory tumors. Secondly, considering the relationship between SBF2-AS1 and drug resistance, SBF2-AS1 provides a new entry point for research on therapeutic tolerance, which is favorable for further clarifying the mechanism of drug resistance from various aspects in cancers. Given this, we maintain that targeting SBF2-AS1 may be an adjunct to conventional drugs to extend the duration of the beneficial effects. However, further experimental studies should be implemented to clarify the detailed drug-resistance mechanisms. In addition to its potential as a therapeutic target, SBF2-AS1 was also detected at high levels in exosomes. Does this imply that SBF2-AS1 can be detected in body fluids (blood, cerebrospinal fluid, etc.) in addition to tissues with differential SBF2-AS1 expression? Therefore, subsequent studied could explore this issue, which will promote early identification of tumorigenesis and thus contribute to treatment at an early stage. Then, the signaling pathways involved in downstream genes of SBF2-AS1 and the interaction of SBF2-AS1 with downstream genes should be particularly refined and expanded, which could link this lncRNA to currently known pathways and then concretize the mechanisms regulating tumor development. Moreover, previous studies have revealed that lncRNAs can interact with proteins through multiple approaches, such as regulating mRNA splicing-associated proteins or protein modifications [122, 123]. However, the interaction between SBF2-AS1 and proteins requires further elucidation. Finally, the regulatory mechanisms and functions involved in SBF2-AS1 have been identified in vitro, indicating that these functions, such as the mechanism of metastasis and EMT, have not been confirmed. For confirmation, further studies conducted in a physiological setting are needed.

## Conclusions

The role of lncRNAs in cancers known currently remains a tip of the iceberg, and future studies are required. This paper reviews SBF2-AS1 regulation of tumor cell proliferation, metastasis, invasion, angiogenesis, chemoresistance, radiosensitivity, and the cell cycle through interactions with different molecules (miRNA, DNA or signaling pathway molecules), indicating that SBF2-AS1 has promise in the future as a diagnostic and prognostic biomarker and a prospective therapeutic target for cancers.

## Data Availability

All data are included in this article.

## Abbreviations

ncRNA: Noncoding RNAs
miRNAs: MicroRNAs
lncRNAs: Long noncoding RNAs
ceRNA: Competitive endogenous RNA
EMT: Epithelial-mesenchymal transition
SBF2-AS1: SET binding factor 2-antisense strand 1
MTMR13: Myotubularin‐related protein 13
LC: Lung cancer
PC: Pancreatic cancer
HCC: Hepatocellular carcinoma
CRC: Colorectal cancer
GC: Gastric cancer
ESCC: Esophageal squamous cell cancer
BC: Breast cancer
GBM: Glioblastoma
OS: Overall survive
ccRCC: Clear cell renal cell carcinoma
TNM: Tumor-node-metastasis
NSCLC: Non-small cell lung cancer
PRC2: Polycomb repressive complex 2
H3K27: Lysine 27 on histone H3
ADAM17: A disintegrin and metalloproteinase 17
GRB2: Growth factor receptor-bound protein 2
MBNL3: Muscleblind-like splicing regulator 3
FOXM1: Forkhead box protein M1
TGFBR1: Transforming growth factor beta receptor 1
SCAMP3: Secretory carrier membrane protein 3
CDKN1A: Cyclin-dependent kinase inhibitor 1A
PFN2: Profilin 2
TWF1: Twinfilin-1
XIAP: X-linked inhibitor of apoptosis protein
HDAC3: Histone deacetylase 3
EMS1: Excess microsporocytes 1
TMZ: Temozolomide
NFAT5: Nuclear factor of activated T cells 5
EGFL7: EGF like domain multiple 7
ZEB1: Zinc finger E-box binding homeobox 1
XRCC4: X-ray repair cross complementing 4
ZFP91: Zinc finger protein 91
RRS1: Ralstonia solanacearum 1
FOXA1: Forkhead box A1
CC: Cervical cancer
AML: Acute myeloid leukemia
PTC: Papillary thyroid cancer
CDK14: Cyclin-dependent kinase 14
ASOs: Antisense oligonucleotides
CRISPR/CAS9: Clustered regularly interspaced short palindromic repeats/CAS9
HULC: Highly upregulated in liver cancer
RIP: RNA Binding Protein Immunoprecipitation
RT-qPCR: Real Time quantitative PCR
MMTV: Mouse mammary tumor virus
